# Response of vertebrate scavengers to power line and road rights-of-way and its implications for bird fatality estimates

**DOI:** 10.1038/s41598-020-72059-7

**Published:** 2020-09-14

**Authors:** Joana Bernardino, Regina Bispo, Ricardo C. Martins, Sara Santos, Francisco Moreira

**Affiliations:** 1 REN Biodiversity Chair, CIBIO/InBIO - Centro de Investigação em Biodiversidade e Recursos Genéticos, Universidade do Porto, Campus Agrário de Vairão, 4485-661 Vairão, Portugal; 2 CIBIO/InBIO - Centro de Investigação em Biodiversidade e Recursos Genéticos, Instituto Superior de Agronomia, Universidade de Lisboa, Tapada da Ajuda, 1349-017 Lisbon, Portugal; 3 Departamento de Matemática & Centro de Matemática e Aplicações (CMA), Faculdade de Ciências e Tecnologia, Universidade Nova de Lisboa, 2829-516 Caparica, Portugal; 4 Mediterranean Institute for Agriculture, Environment and Development (MED), Department of Biology, University of Évora, Mitra, 7002-554 Évora, Portugal

**Keywords:** Environmental impact, Behavioural ecology, Biodiversity, Conservation biology

## Abstract

Linear infrastructures, such as power lines and roads, are an important source of bird mortality. However, little is known on the potential effect of these infrastructures on local scavenger guilds, their foraging activity and the resulting bird carcass removal patterns. This is an important source of bias in studies aiming to quantify bird fatalities due to linear infrastructures. We used camera-traps to record scavenger identity and persistence patterns of bird carcasses placed close to linear infrastructure and nearby controls in two Mediterranean agricultural regions. We found that linear infrastructure influence on scavenger identity varied depending on the region. Contrary to expectations, linear infrastructure presence had either none or a positive effect on carcass persistence, meaning that carcasses placed within power line or road rights-of-way were not removed faster than the ones placed in controls. We conclude that linear infrastructure effect on vertebrate scavenging patterns is likely to be region-specific, and that reliable correction factors for carcass removal-bias in bird fatality estimates require site-specific experiments to characterize local scavenging processes.

## Introduction

Linear infrastructures (LI), such as power lines and roads, are ubiquitous elements of most natural landscapes^1^. Despite their importance for human prosperity and quality of life, LI are responsible for a wide range of impacts on wildlife species and their habitats^2,3^, which need to be quantified to inform effective mitigation actions^4,5^.

One of the most common and harmful LI impacts is bird mortality caused by collisions with vehicles and power line wires^6,7^. The quantification of the number of birds killed by these infrastructures relies on well-designed monitoring protocols which typically comprise regular carcass searches. Due to resource and time limitations, these searches are often conducted on a weekly, monthly or even less frequent basis^8,9^. Several studies provide, however, evidence of a significant removal of small-bird carcasses in the first hours or days after fatality events, both in power lines (e.g.^10,11^) and roads (e.g.^12,13^), hence a large proportion of carcasses (or its remains) have low persistence rates and are missed by searchers. To correct for this bias, field experiments have to estimate the proportion of bird carcasses removed by scavengers or any other event (e.g. traffic, decomposition), to obtain accurate avian fatality estimates^14,15^.

Carcass persistence rates are typically evaluated through field experiments where a known number of bird carcasses is distributed along LI rights-of-way and regularly monitored until no detectable remains are left. Nonetheless, to avoid the so-called scavenger swamping (i.e., abnormal carcass removal by scavengers, due to a saturation of the study area with carcasses^16^), it is recommended that carcasses are placed at least 100–500 m distanced apart (e.g.^17,18^). This requirement greatly restricts the number of carcasses to be placed simultaneously within the LI right-of-way, thus technicians have often questioned the possibility of distributing carcasses in nearby areas (with similar habitats) to achieve the optimal sample size. These alternative sites may not, nonetheless, recreate entirely the conditions of LI rights-of-way, with unknown consequences for the accuracy of the scavenging–bias correction factor derived.

Comprehensive understanding of the scavenging process and its overall effect on carcass persistence is, therefore, crucial for an accurate assessment of LI-related bird mortality levels and for setting adequate monitoring programmes^19,20^. Carcass size^10,21,22^, season or weather conditions^12,23,24^, biogeographic context and microhabitat^11,17,25^ are among the most commonly mentioned factors that influence carcass persistence. Complex interactions between these factors have been shown to modulate vertebrate scavenger communities and their scavenging efficiency^26–28^. The latter depends on (i) the amount of time a carcass takes to be detected by a scavenger; (ii) the way the carcass is consumed by the scavenger (i.e., whether it is completely removed, or more likely to be consumed in situ, often leaving detectable remains behind); and, ultimately, (iii) the amount of time these carcass remains (e.g. feather spots) persist in the field, until they are no longer detectable by searchers.

There is, however, limited understanding of how the presence of the LI itself shapes the composition of the local scavenger guilds, their scavenging efficiency and its cascading effects on carcass persistence (but see^29–31^). Local scavenger guilds and their foraging behaviour are greatly influenced by environmental factors, such as landscape features, weather conditions and carrion availability (e.g.^32–35^). Because of the latter, there is growing belief that scavenging rates within power line and road rights-of-way are higher compared to the surroundings. This claim is mostly driven by the idea that generalist species soon associate the power line or road rights-of-way with a readily available source of food^36–38^. Raptors and corvids often use power lines (including the ones that often run parallel to roads) as perching sites, which may increase their ability to detect and use carrion^39,40^. Likewise, medium-sized carnivores often use anthropogenic linear features, like power line clearings and road verges, for foraging and/or travel^41–43^.

Previous studies showed that some opportunistic vertebrate species actively search for carrion or prey within LI rights-of-way, particularly along roads (e.g.^29,37,44^). However, it has not been properly investigated whether the vertebrate scavenger guilds and their feeding behaviour change under LI influence (compared to control areas), and whether these potential differences in scavenger identity and activity indeed lead to lower persistence of LI-related bird carcasses. In fact, it can also be hypothesized that predation rates may be reduced within LI rights-of-way as vertebrate scavengers may avoid these areas because of potential disturbance (by vehicle traffic in the case of roads) and exclusion effects^30,45–47^.

Here, we investigate the effect of two types of LI, namely transmission power lines and roads, on vertebrate scavenging patterns in two Mediterranean agricultural landscapes, by comparing scavenger identity and carcass persistence under LI influence with nearby control areas. Moreover, we examine if the observed patterns are explained by differences in scavenger efficiency, namely if scavenger identity determined (i) carcass detection (i.e. elapsed time until the first scavenging event); (ii) the likelihood of detectable remains being present after the scavenging event; and, ultimately, (iii) carcass persistence (i.e. elapsed time until the carcass or its remains are no longer detectable).

## Methods

### Study areas

**Figure 1:**
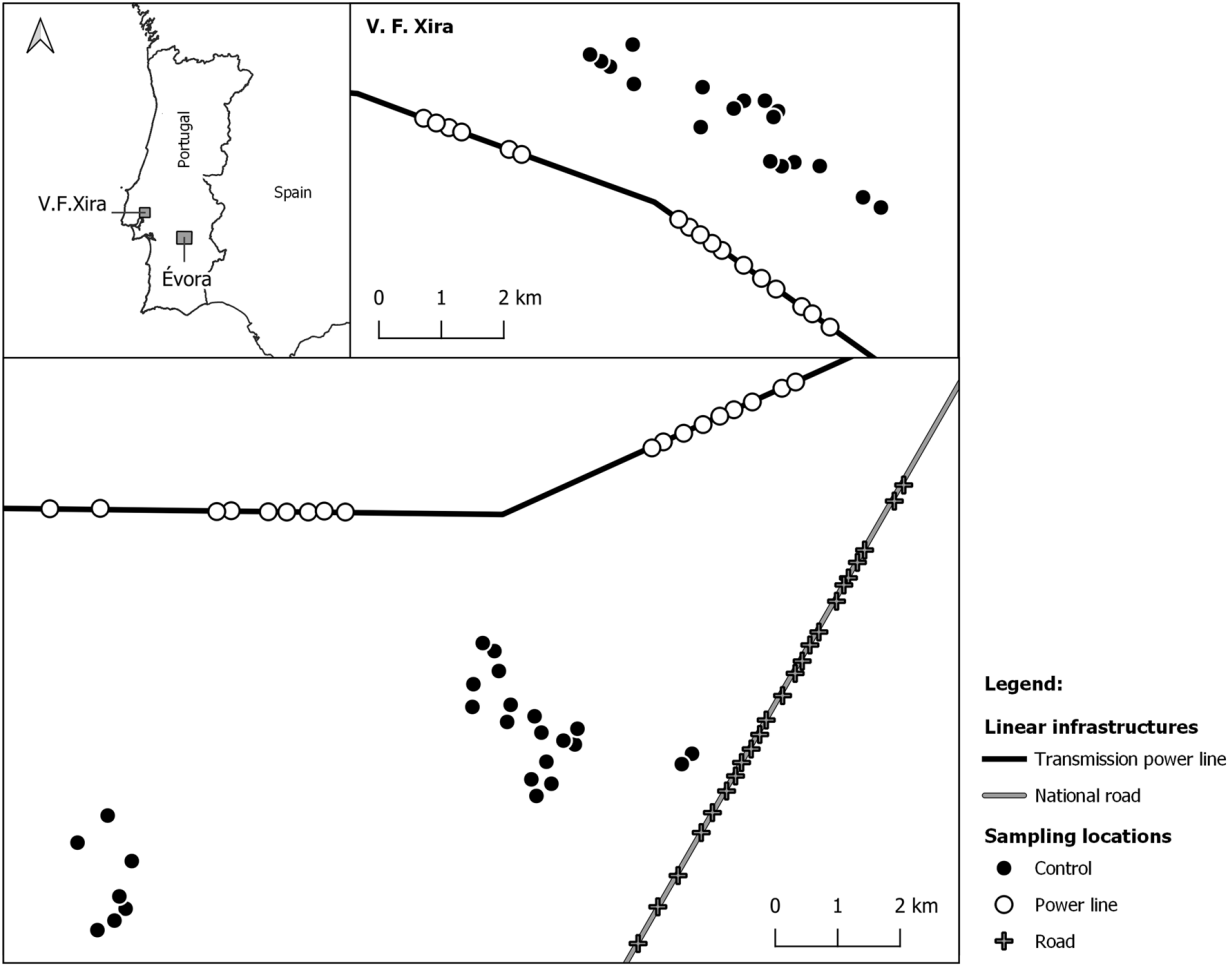
Sampling locations of carcass persistence experiments conducted in V. F. Xira and Évora regions, during the winter periods of 2017 and 2018, respectively. Sampling locations separated less than 200 m apart were never used simultaneously.

The study was conducted in two agricultural landscapes in Central/Southern Portugal, with different management regimes and distanced ca. 90 km apart (Fig. 1).

The first study area (V. F. Xira) is located within a relatively intensive agricultural area, surrounding the Tagus Estuary. Land use is dominated by irrigated rice fields, summer horticultural and cereal crops (mainly tomatoes, melons and maize) and livestock pastures. This study area also comprises a dense network of artificial water channels and non-asphalted roads, a main National road and several transmission and distribution power lines.

The second study area (Évora) is located in a far less intensive farmland. It is dominated by an agro-silvo-pastoral system formed by open Cork oak (*Quercus suber*) and Holm oak (*Q. rotundifolia*) woodlands with natural or cultivated pastures in the undercover (so-called Montados), interspersed with permanent pastures and dry cereal crop-fallow lands, often used for extensive grazing practices. This study area has a lower density network of roads and power lines, compared to the ones of V. F. Xira region.

### Experimental set-up

To control for seasonal variations on carrion removal, we implemented carcass persistence experiments during the 2017 and 2018 winter periods (from January to mid-April) at V. F. Xira and Évora regions, respectively. During the experiments, the average daily temperature was 14.1 °C [7.1–23.6] in V. F. Xira and 10.9 °C [3.5–19.9] in Évora; the monthly average precipitation was, respectively, 66.1 mm [4.7–97.8] and 94.9 mm [48.5–172.5]^48,49^.

In both regions, the experiments were conducted along a PL right-of-away (PL treatment) and in a nearby reference (control) area. In Évora region, the experiment further included the right-of-way of a two-lane paved road (Road treatment), with relative low-traffic volume (ca. 3,700 vehicles per day and ca. 500 vehicles per night, on average^50^) . Power lines included in both experiments were transmission lines (> 110 kV), supported by metal-lattice towers with two parallel shield wires on top, but distinct arrangement of conductors and total height (V. F. Xira: 400 kV double circuit, with 6 conductors arranged in three levels, tower height = 55–60 m; Évora: 150 kV single-circuit; with 3 conductors arranged in a single level, tower height = 30–35 m). Control locations were located at least 500 m from paved roads and transmission power lines and 200 m from distribution (≤ 60 kV) power lines.

We placed 250 free-ranged quail (*Coturnix coturnix*) carcasses (50 carcasses per region and treatment level) purchased from a local breeder. All carcasses were kept frozen from the moment they died until the day of placement and always handled using gloves to avoid traces of human scent. PL carcasses were placed below the power line wires to simulate natural collisions; whereas, road carcasses were placed on the road verge (but ≤ 5 m from road surface limits) to avoid carcass depletion by passing vehicles. Each carcasses was monitored with motion activated infrared cameras (Cuddeback Long Range IR 1224 or LTL ACORN 3310A). Cameras were set up to take time-lapse photos every hour and three sequential photos when triggered by motion, with a forced timeout delay of 1 min between trigger events. Each carcass was monitored up to a maximum of 21 days, or until it was removed by scavengers or decay to the point that we could not detect them (i.e., when we could not locate body parts containing flesh or bone, or ≥ 10 disarticulated feathers^20^). Every camera was visited once a week to confirm carcass presence (or any detectable remains), replace camera batteries, and swap memory cards (to prevent data loss due to camera theft). Whenever a carcass was removed, the retrieved camera was relocated to a new sampling location and a new carcass placed. The same sampling location was not reused unless more than 7 days had passed since the complete removal of the previous carcass.

Carcass locations, within each treatment and control area, were selected in order to guarantee that carcasses were distanced apart at least 200 m, to avoid a potential saturation of the area with carrion^17,25^, and distributed evenly among land uses (V. F. Xira: livestock pastures, horticultural crops and cereal crops; Évora: montado and fallow fields). The proportion of carcasses placed in each land use was not exactly the same across treatment and control areas (Supplementary material, Table S1). To exclude the possibility of confounding effect caused by land use (e.g. one land use being more abundant in controls than in PL or Road treatments), potential differences in land uses among treatments were assessed through a Pearson's Chi-square test, with p-values determined by Monte Carlo simulations (2000 replicates). We found no evidence of significant differences in land uses among treatments (V. F. Xira: ${\upchi }^{2}$ = 4.38, p = 0.122; Évora: ${\upchi }^{2}$ = 0.213, p = 0.941). Since the experiments were performed during winter periods, all carcass locations were characterized by short-grass vegetation, typically with a large amount of bare soil. The undercover gradually became denser throughout the season in all land uses, but never to the point where carcass visibility from the air was significantly compromised. Forest cover was absent in all land uses except in Montado (Évora region), where the tree density was also relatively low.

### Data analysis

We examined all motion-activated and time-lapse photos to determine, for each carcass: (1) the elapsed time until a scavenging event; (2) the vertebrate species responsible for the scavenging event; (3) the presence/absence of detectable carcass remains after the scavenging event, and (4) the elapsed time until the carcass or its remains were no longer detectable. We considered the scavenging events to be independent if more than 30 min had elapsed between consecutive photos of the same scavenger species at the same location. Camera/carcass weekly checks were used to confirm presence/absence of detectable carcass remains (after a scavenging event) and/or time elapsed until remains were no longer detectable, whenever that information could not be extracted from the time-lapse photos. For the analyses, we clustered scavenging species into four main groups: “Raptors”, “Corvids”, “Carnivores” and “Domestic animals”. Although we observed the presence and activity of invertebrate scavengers and rodents (on 5 occasions), these groups were excluded from the analyses since cameras (due to limitations in the sensors) did not capture these events consistently.

All statistical analyses were conducted in R v. 3.6.1^51^. The proportion of scavenging events carried out by each scavenger group, in LI treatments and control areas, was compared using the Pearson's Chi-square test, with p-values determined by Monte Carlo simulations (2000 replicates) (using the function $Boot$ from R-package $car$).

To investigate differences in carcass detection (i.e., elapsed time until the first scavenging event) and carcass persistence (i.e., elapsed time until the carcass or its remains were no longer detectable), we used survival analyses (using the R-package $survival$), which can handle interval- and right-censored time-to-event data^52^. Recorded detection and persistence times were frequently either interval-censored, due to camera trap failure to record the exact scavenging time, or right-censored, because some carcasses persisted until the end of the experiments. We fitted accelerated failure time (AFT) models using the parametric distribution (among the ones most commonly used in carcass persistence models, namely, exponential, Weibull, log-logistic and log-normal^53,54^) that best described carcass detection/persistence times, based on Akaike Information Criterion (see Supplementary material, Table S2, S3, S4 and S5). To assess LI effect on carcass persistence, we fitted separate AFT models for the V. F. Xira and Évora regions, both including ‘Treatment’ (Control, Power line, Road) as an explanatory variable. To investigate differences in scavenger efficiency, we modelled the two response variables (carcass detection and carcass persistence) as function of the explanatory variable ‘Scavenger_group’ (Raptors, Corvids, Carnivores, Domestic animals). Both AFT models also included ‘Treatment_ID’ (with categories V. F. Xira/Control, V. F. Xira/Power line, Évora/Control, Évora/Power line and Évora/Road) as a frailty term (equivalent to the inclusion of a random effect^55^), to account for non-independence between carcasses within the same region and treatment level. To test the overall significance of the explanatory variables, we compared full models against the corresponding null models using a likelihood ratio test (see Supplementary material, Table S6 and S7). The accuracy of the parameters obtained for each final model was evaluated based on 2,000 boot-resamples of the original dataset (using the function $Boot$ from R-package $car$). To visualize the results, carcass persistence curves and restricted mean detection/persistence times (± standard error, s.e.) were estimated non-parametrically using the Kaplan–Meier estimator^56^.

To determine whether the presence/absence of detectable carcass remains after the first scavenging event was significantly different from chance level (probability of 0.5), we performed exact binominal tests, separately for each scavenger group (using the function ${\text{binom}}.{\text{test}}$ from R-package $stats$).

## Results

In total, we recorded 256 independent scavenging events (Table 1). At least 14 species of vertebrate scavengers exploited the monitored quail carcasses (8 species in V. F. Xira, and 11 species in Évora), with Red fox (*Vulpes vulpes*) being the most common scavenger in both regions. The identity of the scavenger was unknown for 77 scavenging events (30.1%), mostly due to camera failure (e.g. humidity and condensation on the lens). From a total of 250 carcasses monitored, only six (12.0%) were never scavenged by vertebrate species during the 21-day experiments. On average, carcasses took 4.60 ± 0.36 days to completely disappear, i.e., to reach to the point at which no carcass parts remained to be detected.

**Table 1:** Number (and percentage) of independent scavenging events detected by vertebrate species for each treatment during the carcass persistence experiments conducted in V. F. Xira and Évora regions (winter periods of 2017 and 2018, respectively).

Scavenger group/species	V. F. Xira		Évora			Total
	CO	PL	CO	PL	RO	
**Raptors**	**21 (42%)**	**4 (8%)**	**2 (4%)**	**1 (2%)**	**2 (4%)**	**30 (11.7%)**
*Buteo buteo*	3 (6%)	2 (4%)	–	1 (2%)	1 (2%)	7 (2.7%)
*Circus aeruginosus*	16 (32%)	2 (4%)	–	–	–	18 (7.0%)
*Circus cyaneus*	1 (2%)	–	–	–	–	1 (0.4%)
*Falco tinnunculus*	1 (2%)	–	–	–	–	1 (0.4%)
*Milvus milvus*	–	–	2 (4%)	–	1 (2%)	3 (1.2%)
**Corvids**	**3 (6%)**	**11 (22%)**	**6 (12%)**	**6 (12%)**	**3 (6%)**	**29 (11.3%)**
*Corvus corax*	–	–	1 (2%)	1 (2%)		2 (0.8%)
*Corvus corone*	3 (6%)	11 (22%)	2 (4%)	1 (2%)	1 (2%)	18 (7.0%)
*Pica pica*	–	–	3 (6%)	4 (8%)	2 (4%)	9 (3.5%)
**Carnivores**	**9 (18%)**	**27 (55%**)	**34 (65%)**	**21 (40%)**	**19 (36%)**	**110 (43.0%)**
*Genetta genetta*	–	–	3 (6%)	5 (10%)	1 (2%)	9 (3.5%)
*Herpestes ichneumon*	–	2 (4%)	–	5 (10%)	–	7 (2.7%)
*Martes foina*		–	6 (12%)	–	4 (8%)	10 (3.9%)
*Vulpes vulpes*	9 (18%)	25 (51%)	25 (48%)	11 (21%)	14 (26%)	84 (32.8%)
**Domestic animals**	**1 (2%)**	**–**	**–**	**1 (2%)**	**8 (15%)**	**10 (3.9%)**
*Canis lupus familiaris*	–	–	–	1 (2%)	–	1 (0.4%)
*Felis catus*	1 (2%)	–	–	–	8 (15%)	9 (3.5%)
**Unknown**	**16 (32%)**	**7 (14%)**	**10 (19%)**	**23 (44%)**	**21 (40%)**	**77 (30.1%)**
Total	50 (100%)	49 (100%)	52 (100%)	52 (100%)	53 (100%)	256 (100%)

Treatment: *CO* control, *PL* power line, *RO* road.

### LI effect on scavenger identity and carcass persistence

**Figure 2:**
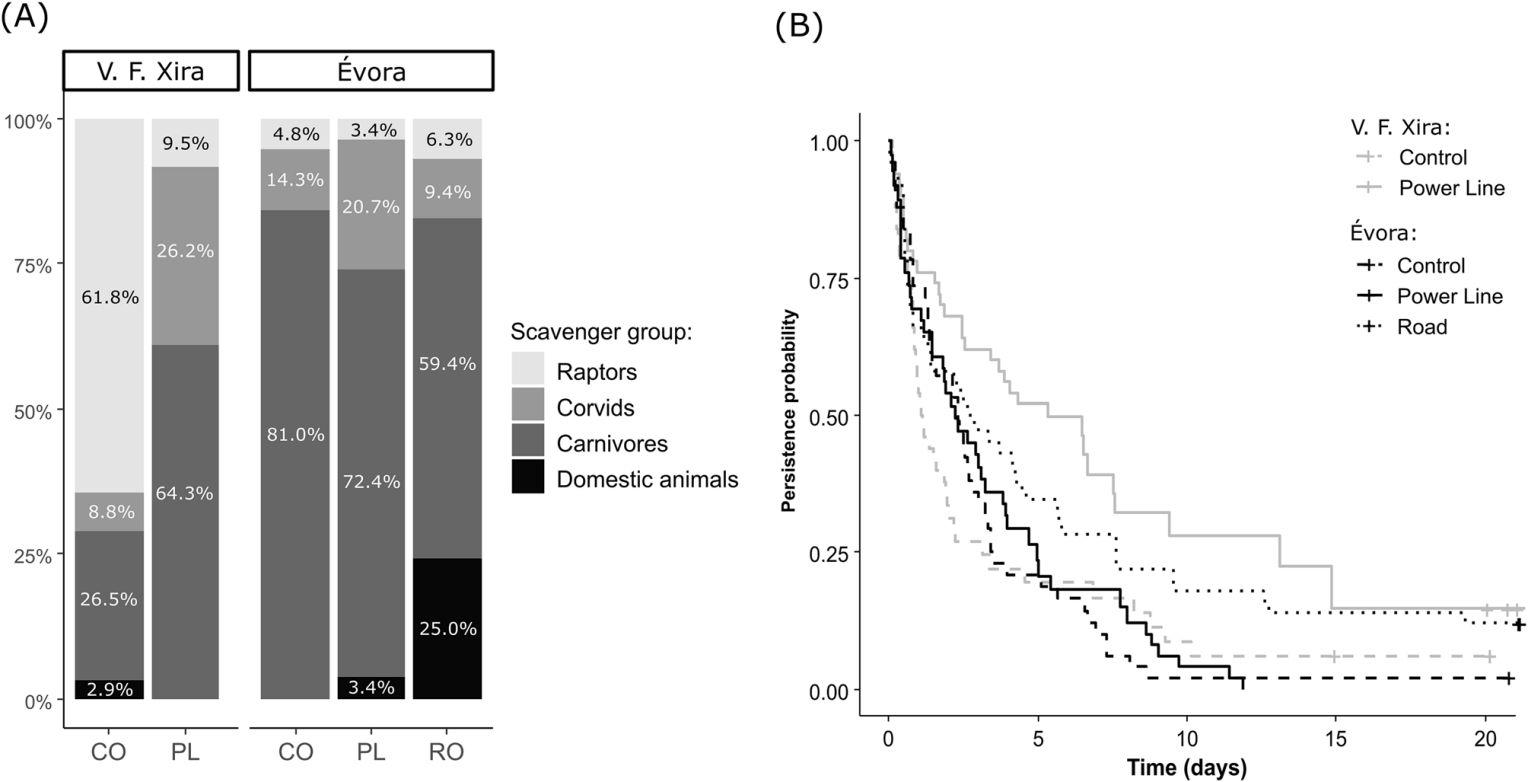
Linear infrastructure effect on (**A**) the proportion of scavenging events carried out by each scavenger group and (**B**) the carcass persistence probability, in V. F. Xira and Évora regions. Treatment: *CO* control, *PL* power line, *RO* road.

The identity of the scavenger species that used the carcasses varied among LI treatments within the same region (Fig. 2A). In V. F. Xira, the proportion of scavenging events carried out by each scavenger group varied noticeably between control and PL locations (${\upchi }^{2}$ = 24.9, df = 2, p < 0.001). Within PL right-of-way, cameras captured the carcasses being mostly scavenged by Carnivores (64.3%), followed by Corvids (26.2%) and Raptors (9.5%); whereas, in control locations, the majority of the carcasses were scavenged by Raptors (61.8%), followed by Carnivores (26.5%) and Corvids (8.8%). In contrast, the proportion of scavenging events carried out by each scavenger group did not differ significantly between control and PL locations in Évora (${\upchi }^{2} \,$ = 3.28, df = 3, p = 0.39), with most of the scavenging being carried out by Carnivores (72.4–81.0%), followed by Corvids (14.3–20.7%) and Raptors (3.4–4.8%). Nonetheless, the proportion of scavenging events carried out by each scavenger group differed significantly between control and Road locations (${\upchi }^{2} \,$ = 10.72, df = 3, p < 0.006), where Domestic animals were responsible for 25.0% of the scavenging events, with a consequent decrease in the proportion of carcasses scavenged by Carnivores (59.4%) and Corvids (9.4%).

Carcass persistence (i.e., elapsed time until the carcass or its remains were no longer detectable) was also significantly affected by LI treatment (V. F. Xira: ${\upchi }^{2} \,$ = 10.6, df = 1, p = 0.0011; Évora:${\upchi }^{2}$ = 10.4, df = 2, p = 0.0056), but differently depending on the region (Table 2). In V. F. Xira, PL presence was associated with higher carcass persistence (mean time: 7.32 ± 0.96 days), when compared to control locations (mean time: 3.32 ± 0.71 days) (Fig. 2B). This pattern was not observed in Évora, where carcass persistence was not significantly affected by PL presence (mean time: 3.37 ± 0.46 days), compared to control locations (mean time: 3.07 ± 0.47 days). On the other hand, carcasses placed on road verges (Évora) tended to persist longer (mean time: 5.60 ± 0.93 days), than the ones placed below the PL or at controls.

**Table 2:** Parameters and bootstrap results of best accelerated failure time models to predict (A) carcass persistence as function of power line or road treatment in V. F. Xira and Évora regions (models A1 and A2, respectively), and (B) carcass detection and carcass persistence as function of scavenger group (model B1 and B2, respectively).

Model	Response variable	Explanatory variable	Coefficient		p-value		
			Original	Bootstrap Median [95% CI]	Original	Bootstrap Median [95% CI]	Signif
(A1)	Carcass persistence	**V. F. Xira/Treatment**					
		*Intercept*	0.33	0.31 [− 0.04 to 0.79]			
		Power line	1.07	1.08 [0.41 to 1.67]	< 0.001	< 0.001 [< 0.001 to 0.31]	***
(A2)	Carcass persistence	**Évora/Treatment**					
		*Intercept*	1.07	1.07 [0.78 to 1.47]			
		Power line	0.04	0.05 [− 0.42 to 0.44]	0.853	0.507 [0.527 to 0.999]	
		Road	0.72	0.72 [0.14 to 1.28]	0.004	0.003 [< 0.001 to 0.634]	**
(B1)	Carcass detection	**Scavenger group**					
		*Intercept*	0.49	0.51 [0.13 to 0.88]			
		Corvids	0.35	0.24 [− 0.17 to 1.26]	0.213	0.298 [< 0.001 to 0.884]	
		Carnivores	0.75	0.71 [− 0.33 to 1.19]	< 0.001	0.002 [< 0.001 to 0.158]	***
		Domestic animals	1.01	0.92 [0.16 to 2.05]	0.011	0.028 [< 0.001 to 0.518]	**
(B2)	Carcass persistence	**Scavenger group**					
		*Intercept*	1.37	1.38 [0.81 to 1.94]			
		Corvids	0.04	0.02 [− 0.71 to 0.80]	0.889	0.439 [0.705 to 0.999]	
		Carnivores	0.05	0.04 [− 0.53 to 0.67]	0.847	0.479 [0.527 to 0.998]	
		Domestic animals	1.25	1.24 [0.27 to 2.70]	0.014	0.019 [< 0.001 to 0.719]	*

Best Accelerated Failure Time (AFT) models selected based on the lowest Akaike Information Criteria (AIC) statistic (see Supplementary material). In AFT models, the coefficients are logarithms of ratios of survival times, so higher (positive) coefficients mean longer times to carcass detection or overall persistence. Reference levels: ‘Control’ for models (A); ‘Raptors’ for models (B). Significance levels: *p < 0.05, **p < 0.01, and ***p < 0.001.

### Scavenger efficiency

**Figure 3:**
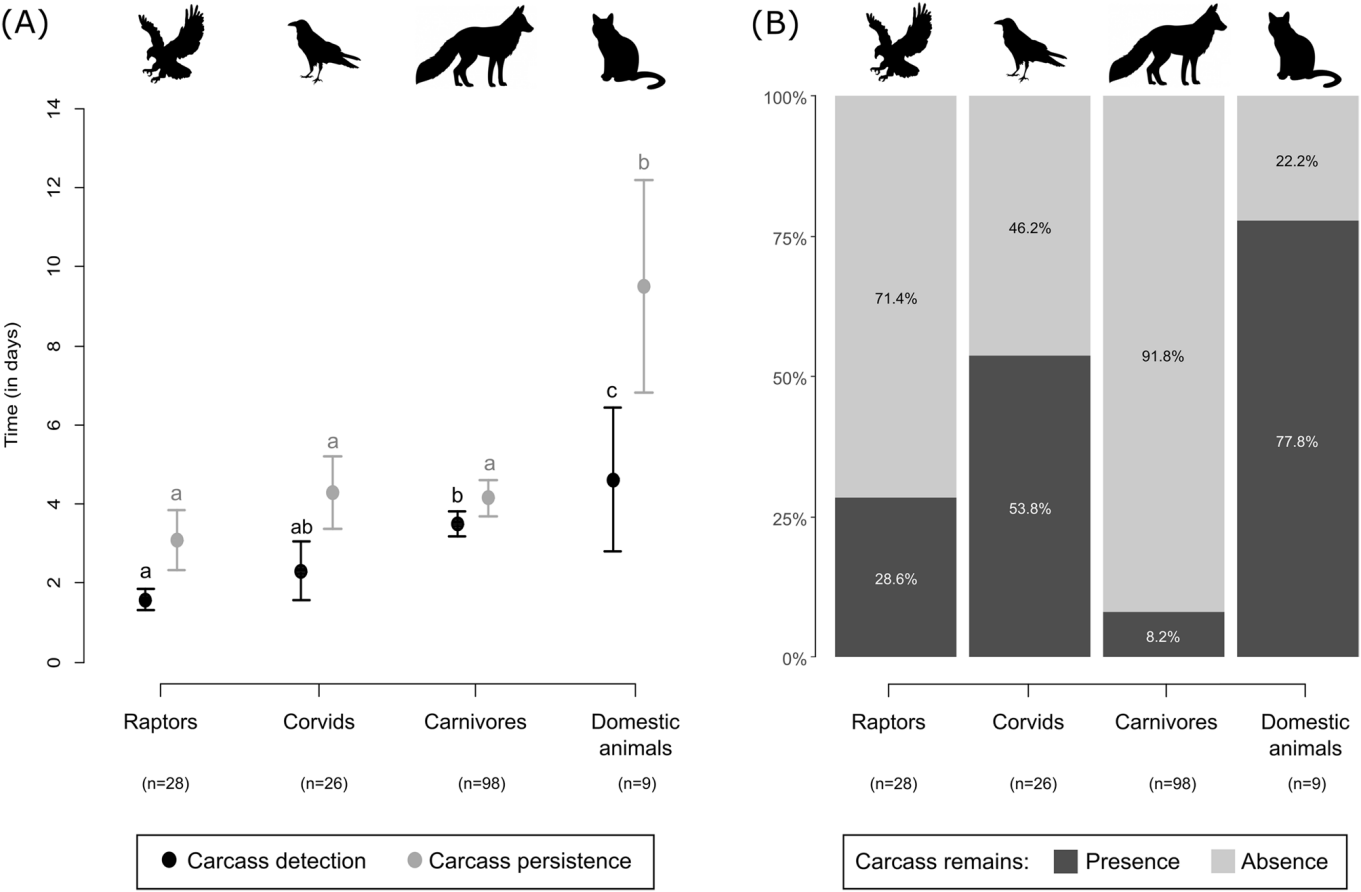
Scavenger identity effect on (**A**) the mean (± standard error) carcass detection and persistence time, and (**B**) proportion of cases with presence vs. absence of detectable carcass remains after the first scavenging event. Whiskers topped by different letters indicate significant differences (p < 0.05) between scavenger groups in carcass detection (black letters) and carcass persistence (grey letters). Number of scavenging events for each scavenger group is contained in parentheses.

Carcass detection (i.e. elapsed time until the first scavenging event) varied significantly among scavenger groups (${\upchi }^{2} \,$ = 18.4, df = 3, p < 0.001; Table 2). In general, avian scavengers tend to detect the carcasses sooner (mean time: 1.93 ± 0.39 days) than mammalian ones (mean time: 3.59 ± 0.34 days) (Fig. 3A).

After the first scavenging event, most of the sampling locations (77.0%) were left without any evidence of the carcass. However, the presence of detectable carcass remains depended on the scavenger group (Fig. 3B). Detectable remains (hereafter denoted as DR) were significantly less likely to be present if the carcass was scavenged by Raptors ($P(DR)$ = 0.25, 95% CI 0.11–0.45; Exact binomial test: p = 0.012) or Carnivores ($P(DR)$ = 0.08, 95% CI 0.04–0.15; Exact binomial test: p < 0.001). For Corvids, the likelihood of leaving carcass remains after the scavenging event was not different from random ($P(DR)$ = 0.53, 95% CI 0.33–0.73; Exact binomial test: p = 0.845). Domestic animals showed a higher probability of leaving carcass remains ($P(DR)$ = 0.67, 95% CI 0.30–0.92), although it was not significantly different from chance level (0.50) (Exact binomial test: p = 0.508).

Overall, carcass persistence (i.e., elapsed time until the carcass or its remains were no longer detectable) also varied among scavengers groups (${\upchi }^{2} \,$ = 48.3, df = 3, p < 0.001), although with a different pattern from the observed for carcass detection (Table 2). Compared to the first model, there were no longer significant differences between carcasses first scavenged by birds (Raptor or Corvids) and Carnivores (mean time: 3.98 ± 0.36 days), but carcasses scavenged by Domestic animals (cats and dogs) still tended to persist significantly longer (mean time: 9.51 ± 2.69 days) (Fig. 3A).

## Discussion

We found that carcasses were detected and removed by scavengers at high rates in both our study regions. This pattern of rapid scavenging has previously been observed in road verges and power lines that bisect agricultural landscapes (e.g.^10–12^), as well as for other anthropogenic sources of bird mortality, like wind farms, fences and pesticides (e.g.^21,25,57^).

### LI effect on scavenger identity and carcass persistence

Several studies showed that both avian and mammal scavengers actively forage within LI rights-of-way due an increased likelihood of finding carrion (e.g.^29,37,44^). Yet, few have investigated whether carcass persistence rates are reduced indeed, particularly in the case of power lines. In our study, the presence of the PL had either none or a positive effect on carcass persistence. In Évora region, there were no significant differences between the PL treatment and control areas, with carnivores being the most common scavengers in both cases. Hill et al*.*^31^, which performed a similar carcass persistence experiment during winter but in forested habitats, also observed almost no differences in scavenger species identity, as well as in the proportion of carcasses scavenged within power line clearings, compared to control areas. A possible explanation, hypothesized by Borner et al*.*^11^, is that scavenger behaviour may be less predictable under power lines because mortality due to collision with wires may be less frequent than mortality on roads.

In V. F. Xira region, however, a completely different scavenging pattern was observed, with the proportion of scavenging events carried out by each scavenger group being significantly different between control and PL locations. Western marsh harriers (*Circus aeruginosus*), abundant in this study region^58^, removed a large proportion of carcasses placed in the control area, at very high rates (see next sub-section, “Scavenger efficiency”), likely leaving few carcasses for carnivores or other scavengers. In turn, scavenging within the PL right-of-way was mostly carried out by carnivores, and at slower rates, most probably due to the reduced number of scavenging events (n = 2) by marsh harriers*.* Unlike other sympatric raptors (e.g. buzzards), that often use PL and other structures as hunting perches, marsh harriers forage primarily through cruising flights^59^, which may explain why the species did not take advantage of PL right-of-way. A similar response to PL presence was noted by DeGregorio et al*.*^60^, that investigated how different landscape features influenced the risk of avian nest predation. These authors observed that, although raptors and crows occurred in high densities near power lines (compared to control areas), raptors were more likely to prey on nests away from power lines.

Because roads are seen as a readily available source of carrion^36–38^, we expected carcass removal rates to be more pronounced within road rights-of-way (even after controlling for carcass removal caused by traffic or other environmental factors). This common assumption was, however, contradicted by the carcass removal patterns observed in Évora region, with carcasses placed on road verges taking more time to be removed compared to carcasses in control locations. This same assumption has also been partially refuted by Lambertucci et al*.*^29^, which investigated the foraging space use of an assemblage of diurnal scavenging raptors in relation to distance from roads in northwest Patagonia. In general, carcasses near roads were detected and removed by diurnal scavenging raptors much faster, but some raptor species (condors and buzzard-eagles) preferred to feed further away from roads, even though they flew all over the area. Hill et al*.*^31^ also found that the scavenger community overall did not show a substantial response to roads and that the proportion of carcasses removed, as well as scavenger arrival time, were similar to controls. In our case, the higher persistence rates near roads were associated to a higher proportion of scavenging events carried out by domestic animals, which tended to remove the carcasses more inefficiently (see next sub-section, “Scavenger efficiency”).

### Scavenger efficiency

Small- and medium-sized carcasses, like the ones tested in this study (quails), are often used by a diverse assemblage of facultative scavengers, i.e., vertebrate predators that opportunistically scavenge on fresh carrion when available^35,61^. Indeed, in our study, most scavenging events were carried out by meso-predators, with red foxes being responsible for almost half of the scavenging events recorded (V. F. Xira: 44.7%; Évora: 48.5%). Although the overall proportion of carcasses consumed by raptors and corvids was smaller (16.8% and 16.2%, respectively), both groups tend to detect the carrion sooner (lower times to first scavenging event) than other groups, probability because avian predators travel long distances while foraging and use their visual perception to find carrion, which is particularity effective in open habitats^35,62^. Conversely, mammal scavengers rely mostly on their olfactory perception to locate carrion and use the decomposition odour as their dominant stimulus, which takes longer to become marked in winter periods^26,63^.

Several studies found a positive relationship between carcass size and persistence rates, as result of the inability of some scavengers to deal with large carcasses and because small carcasses can be exploited by a greater number of species (e.g.^20–22,27^). In our study, carcasses were relatively small and weighted only 100 g, therefore they could easily be removed by medium-sized scavengers. The way carrion was consumed differed, however, among scavenger groups. Carnivores and raptors tend to remove the carcass completely, while corvids and domestic animals (cats and dogs) had equal or higher probability of leaving carcass parts or feather piles behind, as a result of consuming the carcass in situ. Previous studies support the idea that meso-carnivores and diurnal raptors tend to remove carrion more efficiently, while ravens and magpies forage in groups and prefer to take small pieces of the carcass, leaving feather piles or other carcass remains scattered around the site^62,64^. Carcass persistence experiments conducted in urban environment by Hager et al*.*^65^ and Riding and Loss^27^ revealed, however, different scavenging behaviours. In both studies, raccoons, opossums and corvids were observed consuming small avian carcasses resulting from window-collisions mainly at the initial site, whereas cats and squirrels carried carcasses away. These contradicting results suggest that the feeding behaviour within a same scavenger group, particularly across medium-sized carnivores, may vary greatly depending on the species, season and landscape.

In our study, carnivores (mostly red foxes) consistently left no remains of the carcass after the predation event, but took longer to detect the carrion. Thus, the overall persistence time estimated for carcasses scavenged by carnivores was not significantly different from that estimated for carcasses scavenged by corvids (which find carcasses faster but are likely to leave carcass remains behind) or even by raptors (which also find carcasses quickly and leave almost no scavenging evidence). Still, and overall, carcasses scavenged by carnivores and corvids showed higher persistence times (mean time 4.29 ± 0.38 days) compared to raptors (3.08 ± 0.75 days), which explains the higher persistence probability observed within the PL right-of-way in V. F. Xira region. In this study region, these two groups together were responsible for the majority (90.5%) of the scavenging events in the PL right-of-way, while, in the control area, carcasses were predominantly scavenged by raptors (61.8%). Domestic animals, on the other hand, tend to detect the carcass later and, moreover, leave detectable remains behind, which explains the higher carcass persistence rate observed at the road right-of-way in Évora region (where dogs were responsible for a relative high proportion of the scavenging events, compared to PL treatment and controls).

Our experiments were carried out during winter, but seasonal changes in scavenging efficiency are likely to occur (e.g.^11,17,66^). For example, carnivores’ ability to locate carrion is expected to increase with higher temperatures during spring and summer, when olfactory cues are more marked due to decomposers’ activity^26,63^. This can potentially lead to an overall decrease of carcass persistence, particularly in areas where carcasses were mostly consumed by mammals. Moreover, competition for carcasses between avian scavengers, mammalian meso-predators, rodents and invertebrates is expected to increase in warmer months^33,67^.

### Implications for bird fatality estimates

Our research provides new insights on the importance of vertebrate scavenging as a source of bias in bird fatality surveys in LI rights-of-way. Similarly to studies conducted in temperate regions, small-bird carcasses were removed at high rates by vertebrate scavengers, often without leaving a trace of evidence. However, carcass persistence can still vary greatly across regions. For instance, carcass persistence within PL right-of-way was considerably higher in V. F. Xira (mean time 7.32 ± 0.96 days), compared to Évora (mean time 3.37 ± 0.46 days). According to the recently developed “Generalized Estimator” (GenEst) software^54^, in a scenario of weekly carcass searches, the estimated probability of a small carcass persisting until the day of the search is 0.58 in V. F. Xira, but decreases to 0.43 in Évora. This means that, one would have to multiply the number of bird carcasses found by a factor of 1.7 in V. F. Xira and 2.3 in Évora, to obtain the corresponding bird fatality estimates adjusted for scavenging bias. This emphases the extent to which bird fatality estimates are affected by the magnitude of scavenging-bias correction factor obtained for each individual LI project, and reinforces the need for site-specific experiments that characterize the local scavenging rates.

Our results, combined with previous literature, also show that broad generalizations about power line and road effect on scavenging patterns and their cascading effects on carcass persistence may not be appropriate. LI effect on carcass persistence is likely once again to be region-specific and driven by the identity of the local scavenger species, as well as by their inherent ability to find and efficiently remove (or not) carrion from LI rights-of-way. In our study, for instance, the differences in carcass persistence observed between the PL and control areas in V. F. Xira (located near an important wetland) are most probably explained by the high abundance of marsh harriers in the region and their species-specific response to PL right-of-way. Nevertheless, further research on scavenging ecology under LI influence, may help identify the most prevalent carcass persistence patterns across the different types of LI and land uses.

In our study, and contradicting common assumptions, we found no evidence that the presence of a LI increases carcass removal rates due to scavenging by opportunistic vertebrate species. In both agricultural landscapes, LI effect (if present) was positive for carcass persistence, which means that carcasses placed, for instance, next to roads were not removed faster than the ones placed in controls. This unexpected result suggests that bias in small- to medium-sized bird mortality estimates resulting from scavengers’ activity at roads may be negligible at times, particularly when compared to carcass removal rates driven by high vehicle-traffic^68,69^.

Finally, our findings may also contribute for a better planning and design of future carcass persistence experiments in bird-fatality monitoring programs, particularly the ones conducted in power lines. To comply with a pre-established trial sample size, technicians may feel tempted to saturate the power line right-of-way with carcasses—which may induce scavenger swamping ^20,70^—or, alternatively, to place additional carcasses in nearby locations. Our results show that placing trial carcasses outside the LI right-of-way, even in locations with relatively similar habitat features, may lead to biased persistence rates. For instance, in one of our study regions (V. F. Xira), the use of carcass removal data from the control area to adjust the observed bird casualties within the PL right-of-way (assuming e.g. weekly carcass searches) would have resulted in > 2-fold higher bird fatality estimates. Thus, the implementation of carcass persistence experiments outside the PL right-of-way should be, as much as possible, avoided. The appropriate sample size for estimating carcass persistence should be assured through careful planning of the experiments and by placing carcasses, if necessary, spaced out in time, so scavenger swamping can be avoided^15^.

## Supplementary information

Supplementary Tables.

## Data Availability

The datasets generated during the current study are available from the corresponding author on request.

## References

[1] Dulac, J. *Global land transport infrastructure requirements: estimating road and railway infrastructure capacity and costs to 2050.* (International Energy Agency, Paris, France, 2013).

[2] D’Amico M (2018). Bird on the wire: landscape planning considering costs and benefits for bird populations coexisting with power lines. AMBIO A J. Hum. Environ..

[3] Morelli F, Beim M, Jerzak L, Jones D, Tryjanowski P (2014). Can roads, railways and related structures have positive effects on birds? A review. Transp. Res. Part D Transp. Environ..

[4] Laurance WF (2015). Reducing the global environmental impacts of rapid infrastructure expansion. Curr. Biol..

[5] Ascensão F (2019). Beware that the lack of wildlife mortality records can mask a serious impact of linear infrastructures. Glob. Ecol. Conserv..

[6] Bernardino J (2018). Bird collisions with power lines: state of the art and priority areas for research. Biol. Conserv..

[7] Loss SR, Will T, Marra PP (2014). Estimation of bird-vehicle collision mortality on U.S. roads. J. Wildl. Manag..

[8] Collinson WJ, Parker DM, Bernard RTF, Reilly BK, Davies-Mostert HT (2014). Wildlife road traffic accidents: a standardized protocol for counting flattened fauna. Ecol. Evol..

[9] Barrientos R, Alonso JC, Ponce C, Palacín C (2011). Meta-analysis of the effectiveness of marked wire in reducing avian collisions with power lines. Conserv. Biol..

[10] Ponce C, Alonso JC, Argandoña G, García Fernández A, Carrasco M (2010). Carcass removal by scavengers and search accuracy affect bird mortality estimates at power lines. Anim. Conserv..

[11] Borner L (2017). Bird collision with power lines: estimating carcass persistence and detection associated with ground search surveys. Ecosphere.

[12] Guinard É, Julliard R, Barbraud C (2012). Motorways and bird traffic casualties: carcasses surveys and scavenging bias. Biol. Conserv..

[13] Santos SM, Carvalho F, Mira A (2011). How long do the dead survive on the road? Carcass persistence probability and implications for road-kill monitoring surveys. PLoS ONE.

[14] Barrientos R (2018). A review of searcher efficiency and carcass persistence in infrastructure-driven mortality assessment studies. Biol. Conserv..

[15] Huso M, Dalthorp D, Miller TJ, Bruns D (2016). Wind energy development: methods to assess bird and bat fatality rates post-construction. Hum. Wildl. Interact..

[16] Smallwood KS (2007). Estimating wind turbine-caused bird mortality. J. Wildl. Manag..

[17] Costantini D, Gustin M, Ferrarini A, Dell’Omo G (2017). Estimates of avian collision with power lines and carcass disappearance across differing environments. Anim. Conserv..

[18] Schutgens M, Shaw JM, Ryan PG (2014). Estimating scavenger and search bias for collision fatality surveys of large birds on power lines in the Karoo, South Africa. Ostrich.

[19] Loss SR, Will T, Marra PP (2012). Direct human-caused mortality of birds: improving quantification of magnitude and assessment of population impact. Front. Ecol. Environ..

[20] Smallwood KS, Bell DA, Snyder SA, DiDonato JE (2010). Novel scavenger removal trials increase wind turbine—caused avian fatality estimates. J. Wildl. Manag..

[21] Farfán MA, Duarte J, Fa JE, Real R, Vargas JM (2017). Testing for errors in estimating bird mortality rates at wind farms and power lines. Bird Conserv. Int..

[22] Flint PL, Lance EW, Sowl KM, Donnelly TF (2010). Estimating carcass persistence and scavenging bias in a human-influenced landscape in western Alaska. J. F. Ornithol..

[23] Paula J (2015). Camera-trapping as a methodology to assess the persistence of wildlife carcasses resulting from collisions with human-made structures. Wildl. Res..

[24] Shaw JM, van der Merwe R, van der Merwe E, Ryan PG (2015). Winter scavenging rates under power lines in the Karoo, South Africa. Afr. J. Wildl. Res..

[25] Stevens BS, Reese KP, Connelly JW (2011). Survival and detectability bias of avian fence collision surveys in sagebrush steppe. J. Wildl. Manag..

[26] Turner KL, Abernethy EF, Conner LM, Rhodes OE, Beasley JC (2017). Abiotic and biotic factors modulate carrion fate and vertebrate scavenging communities. Ecology.

[27] Riding CS, Loss SR (2018). Factors influencing experimental estimation of scavenger removal and observer detection in bird-window collision surveys. Ecol. Appl..

[28] Rosene W, Lay DW (1963). Disappearance and visibility of quail remains. J. Wildl. Manag..

[29] Lambertucci SA, Speziale KL, Rogers TE, Morales JM (2009). How do roads affect the habitat use of an assemblage of scavenging raptors?. Biodivers. Conserv..

[30] Donázar JA, Ceballos O, Cortes-Avizanda A (2018). Tourism in protected areas: disentangling road and traffic effects on intra-guild scavenging processes. Sci. Total Environ..

[31] Hill JE, DeVault TL, Beasley JC, Rhodes OE, Belant JL (2018). Roads do not increase carrion use by a vertebrate scavenging community. Sci. Rep..

[32] Huijbers CM (2015). Limited functional redundancy in vertebrate scavenger guilds fails to compensate for the loss of raptors from urbanized sandy beaches. Divers. Distrib..

[33] Olson ZH, Beasley JC, Rhodes OE (2016). Carcass type affects local scavenger guilds more than habitat connectivity. PLoS ONE.

[34] Smith JB, Laatsch LJ, Beasley JC (2017). Spatial complexity of carcass location influences vertebrate scavenger efficiency and species composition. Sci. Rep..

[35] DeVault TL, Rhodes Olin E, Shivik JA (2003). Scavenging by vertebrates: behavioral, ecological, and evolutionary perspectives on an important energy transfer pathway in terrestrial ecosystems. Oikos.

[36] Joseph GS, Seymour CL, Foord SH (2017). The effect of infrastructure on the invasion of a generalist predator: pied crows in southern Africa as a case-study. Biol. Conserv..

[37] Dean WRJ, Milton SJ, Anderson MD (2006). Use of road kills and roadside vegetation by Pied and Cape Crows in semi-arid South Africa. Ostrich.

[38] Slater FM (2002). An assessment of wildlife road casualties—the potential discrepancy between numbers counted and numbers killed. Web Ecol..

[39] Knight RL, Kawashima JY (1993). Responses of raven and red-tailed hawk populations to linear right-of-ways. J. Wildl. Manag..

[40] Meunier FD, Verheyden C, Jouventin P (2000). Use of roadsides by diurnal raptors in agricultural landscapes. Biol. Conserv..

[41] Andersen GE, Johnson CN, Barmuta LA, Jones ME (2017). Use of anthropogenic linear features by two medium-sized carnivores in reserved and agricultural landscapes. Sci. Rep..

[42] Frey SN, Conover MR (2006). Habitat use by meso-predators in a corridor environment. J. Wildl. Manag..

[43] Raiter KG, Hobbs RJ, Possingham HP, Valentine LE, Prober SM (2018). Vehicle tracks are predator highways in intact landscapes. Biol. Conserv..

[44] Silva C, Simões MP, Mira A, Santos SM (2019). Factors influencing predator roadkills: the availability of prey in road verges. J. Environ. Manag..

[45] Bautista LM (2004). Effect of weekend road traffic on the use of space by raptors. Conserv. Biol..

[46] Benítez-López A, Alkemade R, Verweij PA (2010). The impacts of roads and other infrastructure on mammal and bird populations: a meta-analysis. Biol. Conserv..

[47] Tyler N (2014). Ultraviolet vision and avoidance of power lines in birds and mammals. Conserv. Biol..

[48] IPMA. *Boletins Climatológicos Mensais *(*Portugal Continental*). *Instituto Português do Mar e da Atmosfera, I. P. *(*IPMA, I. P.*). https://www.ipma.pt/pt/publicacoes/ (2017).

[49] IPMA. *Boletins Climatológicos Mensais *(*Portugal Continental*). *Instituto Português do Mar e da Atmosfera, I. P. *(*IPMA, I. P.*). https://www.ipma.pt/pt/publicacoes/ (2018).

[50] E.P. *Recenseamento de tráfego *(*2005*)*—distrito de Évora* (Estradas de Portugal, S.A., 2005).

[51] R Development Core Team. R: a language and environment for statistical computing, version 3.6.1 (2019).

[52] Therneau, T. M. A Package for Survival Analysis in S. version 2.44-1.1 (2019).

[53] Bispo R, Bernardino J, Marques TA, Pestana D, LitadaSilva J, Caeiro F, Natário I, Braumann CA (2013). Discrimination between parametric survival models for removal times of bird carcasses in scavenger removal trials at wind turbines sites BT. Advances in Regression, Survival Analysis, Extreme Values, Markov Processes and Other Statistical Applications.

[54] Dalthorp, D. *et al. GenEst statistical models—A generalized estimator of mortality*. *Techniques and Methods *(2018). https://pubs.er.usgs.gov/publication/tm7A2. 10.3133/tm7A2.

[55] Gutierrez RG (2002). Parametric frailty and shared frailty survival models. Stata J..

[56] Kaplan EL, Meier P (1958). Nonparametric estimation from incomplete observations. J. Am. Stat. Assoc..

[57] Linz GM, Bergman DL, Bleier WJ (1997). Estimating survival of song bird carcasses in crops and woodlots. Prairie Nat..

[58] Lourenço PM (2009). Rice field use by raptors in two Portuguese wetlands. Airo.

[59] Simmons RE (2000). Harriers of the World: Their Behaviour and Ecology.

[60] DeGregorio BA, Weatherhead PJ, Sperry JH (2014). Power lines, roads, and avian nest survival: effects on predator identity and predation intensity. Ecol. Evol..

[61] Beasley JC, Olson ZH, DeVault TL, Benbow ME, Tomberlin JK, Tarone AM (2015). Ecological role of vertebrate scavengers. Carrion Ecology, Evolution and Their Applications.

[62] Peisley RK, Saunders ME, Robinson WA, Luck GW (2017). The role of avian scavengers in the breakdown of carcasses in pastoral landscapes. EMU Austral. Ornithol..

[63] DeVault TL, Rhodes OE (2002). Identification of vertebrate scavengers of small mammal carcasses in a forested landscape. Acta Theriol. (Warsz).

[64] Hiraldo F, Blanco JC, Bustamante J (1991). Unspecialized exploitation of small carcasses by birds. Bird Study.

[65] Hager SB, Cosentino BJ, McKay KJ (2012). Scavenging affects persistence of avian carcasses resulting from window collisions in an urban landscape. J. F. Ornithol..

[66] Prosser P, Nattrass C, Prosser C (2008). Rate of removal of bird carcasses in arable farmland by predators and scavengers. Ecotoxicol. Environ. Saf..

[67] DeVault TL, Olson ZH, Beasley JC, Rhodes OE (2011). Mesopredators dominate competition for carrion in an agricultural landscape. Basic Appl. Ecol..

[68] Ratton P, Secco H, da Rosa CA (2014). Carcass permanency time and its implications to the roadkill data. Eur. J. Wildl. Res..

[69] Santos RAL (2016). Carcass persistence and detectability: reducing the uncertainty surrounding wildlife-vehicle collision surveys. PLoS ONE.

[70] Linz GM, Davis JE, Engeman RM, Otis DL, Avery ML (1991). Estimating survival of bird carcasses in Cattail Marshes. Wildl. Soc. Bull..

