# Supplementary material for: Response of vertebrate scavengers to power line and road rights-of-way and its implications for bird fatality estimates
Source: Sci Rep. 2020 Sep 14;10:15014. doi: 10.1038/s41598-020-72059-7 (PMC7490418; doi:10.1038/s41598-020-72059-7)
Supplement: Supplementary file 1 — Supplementary Tables. [file 41598_2020_72059_MOESM1_ESM.pdf]

Response of vertebrate scavengers to power line and road rights-of-way and its implications for bird fatality estimates

Joana Bernardino, Regina Bispo, Ricardo C. Martins, Sara Santos and Francisco Moreira

ELECTRONIC SUPPLEMENTARY MATERIAL

Land use among linear infrastructure treatments and controls

Table S1. Number (and percentage) of carcasses placed by land use across treatment and control areas in V. F. Xira and Évora regions.

Region / Land use	Control	Power line	Road
**V. F. Xira**			
Livestock pastures	19 (38%)	29 (58%)	-
Horticultural crops	13 (26%)	7 (14%)	-
Cereal crops	18 (36%)	14 (28%)	-
**TOTAL**	50 (100%)	50 (100%)	-
**Évora**			
Montado	24 (48%)	24 (48%)	26 (52%)
Fallow fields	26 (52%)	26 (52%)	24 (48%)
**TOTAL**	50 (100%)	50 (100%)	50 (100%)

Selection of best parametric distribution for each accelerated failure time model

Table S2. Selection of best parametric distribution (using Akaike Information Criterion, AIC) for the accelerated failure time model to assess 'LI treatment' effect on carcass persistence in V. F. Xira region.

Distribution	K	AIC	Δ_{AIC}	AIC weight
Log-normal	3	414.70	0.00	0.58
Log logistic	3	415.38	0.68	0.41
Weibull	3	424.29	9.59	0.00
Exponential	2	441.48	26.77	0.00

K – number of parameters; Δ_{AIC} – difference in AIC relative to the highest ranked model.

Table S3. Selection of best parametric distribution (using Akaike Information Criterion, AIC) for the accelerated failure time model to assess LI treatment effect on carcass persistence in Évora region.

Distribution	K	AIC	Δ_{AIC}	AIC weight
Weibull	4	688.15	0.00	0.62
Log-normal	4	690.40	2.25	0.20
Log-logistic	4	691.19	3.04	0.14
Exponential	3	693.76	5.61	0.04

K – number of parameters; Δ_{AIC} – difference in AIC relative to the highest ranked model.

Table S4. Selection of best parametric distribution (using Akaike Information Criterion, AIC) for the accelerated failure time model to assess 'Scavenger group' effect on carcass detection.

Distribution	K	AIC	Δ_{AIC}	AIC weight
Exponential	4	674.09	0.00	0.71
Weibull	5	675.89	1.80	0.29
Log-normal	5	688.11	14.01	0.00
Log-logistic	5	690.48	16.38	0.00

K – number of parameters; Δ_{AIC} – difference in AIC relative to the highest ranked model.

Table S5. Selection of best parametric distribution (using Akaike Information Criterion, AIC) for the accelerated failure time model to assess 'Scavenger group' effect on carcass persistence.

Distribution	K	AIC	Δ_{AIC}	AIC weight
Log-normal	5	715.53	0.00	0.56
Weibull	5	717.02	1.48	0.27
Log-logistic	5	717.95	2.42	0.17
Exponential	4	724.11	8.57	0.01

K – number of parameters; Δ_{AIC} – difference in AIC relative to the highest ranked model.

Results of the Likelihood ratio tests to compare full vs. null accelerated failure time models

Table S6. Results of the Likelihood ratio tests to compare the accelerated failure time models for ‘LI treatment’ effect on carcass persistence in V. F. Xira and Évora region, against the corresponding null models.

Model	Deviance	df	p-value
V. F. Xira	10.60397	1	0.0011
Évora	10.35518	2	0.0056

Table S7. Results of the Likelihood ratio tests to compare the accelerated failure time models for Scavenger group’ effect on carcass detection and carcass persistence, against the corresponding null models.

Model	Deviance	df	p-value
Carcass detection	14.97233	3	0.0018
Carcass persistence	16.52639	3	0.0009
